# The Influence of Ketone Bodies on Circadian Processes Regarding Appetite, Sleep and Hormone Release: A Systematic Review of the Literature

**DOI:** 10.3390/nu14071410

**Published:** 2022-03-28

**Authors:** Davide Masi, Maria Elena Spoltore, Rebecca Rossetti, Mikiko Watanabe, Rossella Tozzi, Alessandra Caputi, Renata Risi, Angela Balena, Orietta Gandini, Stefania Mariani, Giovanni Spera, Lucio Gnessi, Carla Lubrano

**Affiliations:** 1 Department of Experimental Medicine, Section of Medical Pathophysiology, Food Science and Endocrinology, Sapienza University of Rome, 00161 Rome, Italy; davide.masi@uniroma1.it (D.M.); mariaelena.spoltore@uniroma1.it (M.E.S.); rebecca.rossetti@uniroma1.it (R.R.); mikiko.watanabe@uniroma1.it (M.W.); alessandra.caputi@uniroma1.it (A.C.); renata.risi@uniroma1.it (R.R.); angela.balena@uniroma1.it (A.B.); s.mariani@uniroma1.it (S.M.); giannispera@yahoo.com (G.S.); lucio.gnessi@uniroma1.it (L.G.); 2 Department of Molecular Medicine, Sapienza University of Rome, 00161 Rome, Italy; rossella.tozzi@uniroma1.it (R.T.); orietta.gandini@uniroma1.it (O.G.)

**Keywords:** chrononutrition, clock gene, circadian rhythm, ketone bodies, sleep, appetite, hormone regulation

## Abstract

Chrononutrition is an emerging branch of chronobiology focusing on the profound interactions between biological rhythms and metabolism. This framework suggests that, just like all biological processes, even nutrition follows a circadian pattern. Recent findings elucidated the metabolic roles of circadian clocks in the regulation of both hormone release and the daily feeding–fasting cycle. Apart from serving as energy fuel, ketone bodies play pivotal roles as signaling mediators and drivers of gene transcription, promoting food anticipation and loss of appetite. Herein we provide a comprehensive review of the literature on the effects of the ketogenic diets on biological processes that follow circadian rhythms, among them appetite, sleep, and endocrine function.

## 1. Introduction

Chronobiology studies the variations in the timing and duration of biological activities in living organisms, such as animals, plants, bacteria, fungi, and protozoa, as a consequence of their adaptation to solar-related rhythm. Circadian rhythm is a 24-h cycle generated by endogenous clocks that influences several essential biological processes such as neuronal, endocrine and metabolic functions [1]. As suggested by Daan et al., the synchronization of these circadian processes, also defined as “entrainment” is the mechanism that living organisms have to adapt their behavior and physiological functions to the circadian rhythm caused by the rotation of the earth, which defines the external cycle period (T) [2].

In mammals, the master circadian clock is primarily located in the suprachiasmatic nuclei (SCN) of the hypothalamus [3], whose phase is synchronized to light/dark cycles by signal input from the retina [4].

Previous studies on Drosophila and mice by Pittendrigh and Aschoof laid the foundations for canonical theories of chronobiology, hypothesizing that biological clocks and their precision are determined by the intrinsic period (τ) of the circadian master clock [5], but also by environmental temporal factors that can reset the biological clock [6].

The molecular architecture of the mammalian master clock is characterized by the presence of a transcriptional–translational feedback loop. In particular, two transcription factors, CLOCK and BMAL1, stimulate the transcription of Per and Cry genes encoding for proteins that translocate to the nucleus and subsequently repress the transcriptional activation of their own genes [7].

Recent findings have demonstrated that multicellular organisms also have numerous clocks outside the brain, within all major tissues and cells. Circadian rhythmicity is thus generated by complex networks connecting “master clock” and “peripheral clocks”. These peripheral clocks drive local tissue-specific processes, such as hormone secretions and behavioral functions including sleep–wake and feeding–fasting cycles, as demonstrated by both in vitro and in vivo studies [8]. Specifically, the pancreatic clock regulates insulin secretion in relation to meals [9], the hepatic clock regulates glycemic control under fasting conditions [10], the adipose tissue clock regulates lipid metabolism [11], while the skeletal muscle clock regulates glucose metabolism [12].

While light is the main stimulus that regulates circadian clocks, a recent review showed that nutrients, timing of meals and resulting diets can act as effective stimuli for various peripheral pathways [8]. Recently, the effects of meal timing on circadian synchronization and metabolism have been evaluated. It is known that limiting food intake to a short time window can cause behavioral and physiological changes. For example, a key experiment showed that limiting food intake to the middle of the light phase can reduce body weight and improve metabolic health in mice compared to controls fed ad libitum.

Among the most effective of these dietary patterns for weight loss there are the ketogenic diets (KDs), which have received increasing attention in recent years [13], but remain little studied in relation to circadian rhythm [14].

KD is characterized by a very low carbohydrate intake (5–10% of total daily calorie intake, or 20–50 g per day) [15]. Among KDs, high-fat ketogenic diets (HFKD) contain a limited number of carbohydrates < 50 g/day, with ad libitum fat and calorie intake. While HFKD began as a treatment for refractory epilepsy, KD with reduced caloric content is now the most widespread diet for weight loss. Very low-calorie KD (VLCKD), equivalent to protein-sparing modified fast (PSMF), typically involves meal replacements including protein derived from soy, whey, eggs and green peas [16,17]. The broader category of which VLCKDs form a part, very low-calorie diets (VLCD), consists of severely limited calorie intake (400–800 kcal/daily). VLCDs, however, are not necessarily associated with major carbohydrate intake reduction, and as a consequence they do not always induce ketosis [18].

Both HFKD and VLCKD may induce ketosis with ketone bodies (KBs) production. KBs refer to three different acidic chemicals including beta-hydroxybutyrate (BOHB), acetoacetate (AcAc) and acetone (Ac). KBs are continually synthesized, although their hepatic production increases when cells are forced to use primarily fats rather than sugars [19]. Therefore, KBs are formed in large amounts under two different conditions: prolonged fasting and uncontrolled insulin-dependent diabetes. These two conditions are characterized by a lack of glucose in the cells. After being synthesized, KBs circulate in the blood and are utilized by various tissues (heart and skeletal muscles, brain, etc.) or eliminated through urination and exhalation [20]. When an individual is not fasting, KBs play a marginal role in metabolism, whereas during periods of prolonged fasting, they become an important energy source, especially for the brain. When one’s diet does not provide sufficient amount of glucose and the scarce hepatic reserves of sugar are exhausted, gluconeogenesis—which allows ex novo synthesis of glucose from certain amino acids or from glycerol and lactic acid—is activated [21]. Together, ketosis and gluconeogenesis are able to satisfy the energy needs of the brain. If KBs did not exist, an organism would be forced to catabolize important quantities of muscle proteins to meet these energy needs through gluconeogenesis alone. The increased synthesis of KBs that occurs during fasting is therefore a protective adaptive mechanism in case of prolonged food restriction. The extent to which circadian rhythms can be influenced by KBs has not yet been fully elucidated.

There are four different dietary approaches that can lead to a state of ketosis: (1) time-restricted feeding, such as eating in an 8-h window and fasting for the remainder of the day; (2) intermittent fasting, where one or more days of fasting are interspersed with normal ad libitum days of eating; (3) chronic energy restriction, characterized by a significant reduction of caloric intake (up to 40%) and no change in the frequency of meals; (4) significant carbohydrate restriction (<20–50 g/d) [22,23,24]. Only the third and the fourth dietary approaches have been shown to lead definitively to the production of KBs for prolonged periods of time [15]. In addition, there are several other health conditions, besides overweight and obesity, for which the KD is recommended as a first-line treatment, including psychiatric disorders [25], polycystic ovary syndrome (PCOS) [26], and non-alcoholic fatty liver disease (NAFLD) [27,28]. Recent studies have demonstrated that specific types of KDs are safe even in case of chronic kidney disease [17,29] and may be useful in preventing and supporting SarsCoV2 disease [30,31].

The aim of the current work is to provide a summary of the existing evidence on KDs putative impact on a subset of circadian processes: appetite, sleep quality, and endocrine function.

## 2. Materials and Methods

Current literature was systematically reviewed. The research was conducted on PubMed, Embase, and Cochrane by using the following keywords: “ketogenic diet”, “ketone bodies” or “ketosis” AND “chrononutrition”, “sleep”, “appetite”, “hormone”, “testosterone”, “thyroid stimulating hormone”, “prolactin”, “insulin-like growth factor 1”, ”growth hormone”, or “melatonin”.

We included all studies with the following criteria: (1) observational prospective and retrospective studies, case-control studies, cohort studies, randomized clinical trials (RCTs), systematic reviews and meta-analyses; (2) studies without age limitations; (3) studies written in English.

A total of 3419 studies were identified through database searches and reference lists of retrieved articles. After removal of 3327 studies based on title and abstract, 50 full text articles were assessed, and 39 included in the present review.

## 3. Results

### 3.1. KD and Chrononutrition

Although KBs are traditionally viewed as metabolic substrates enlisted only in carbohydrate restriction, numerous studies have elucidated their role as crucial metabolic and signaling mediators. However, little is known regarding their impact on circadian homeostasis. One important finding is that the hepatic rhythmic release of BOHB appears to be implicated in driving food anticipation via feedback to the hypothalamus, as reported by Chavan et al. [32].

Furthermore, it has recently been hypothesized that KBs may also affect peripheral pathways of circadian physiology. In this regard, a study by Tognini et al. revealed that KDs can have profound and differential effects on liver and gut clocks, potentially inducing tissue-specific amplitude of clock-controlled genes and transcription factors, including BMAL1. After feeding 8-week-old C57BL/6 mice with a HFKD for 4 weeks and comparing them to control chow-fed mice, the authors found that the KD abolishes rhythmicity in respiratory metabolism. Moreover, the KD induces circadian oscillation in both serum and intestinal BOHB levels, an occurrence that parallels tissue specific cyclic histone deacetylase (HDAC) activity and histone acetylation [33]. However, the master clock gene expression appears to be resistant to potential perturbations caused by the KD.

### 3.2. Effect of the KD on Appetite Regulation

Hunger and satiety are fundamental variables involved in body weight regulation. Although eating is under voluntary control, food intake and energy expenditure are under the control of the central nervous system, which receives information from adipose tissue, gastrointestinal tract (GIT), and from blood and peripheral sensory receptors.

The hypothalamus is the main responsible for hunger/satiety control, it is a site of convergence and integration of many central and peripheral signals that are involved in food intake and energy expenditure mechanisms including endocannabinoids (ECs), neuropeptide Y (NPY), proopiomelanocortin (POMC), melanin-concentrating hormone (MCH), α-melanocyte stimulating hormone (α-MSH), agouti-related peptide (AgRP), cocaine- and amphetamine-regulated transcript (CART), cholecystokinin (CCK), and glucagon-like peptide 1 (GLP-1) [34].

Among the gastrointestinal hormones, CCK, peptide YY (PYY), and GLP-1 represent satiety signals capable of influencing the arcuate nucleus of the hypothalamus where they promote the POMC/αMSH pathway thus reducing appetite [35].

In contrast, acylated ghrelin is a strong orexigenic molecule produced both in the stomach and in the hypothalamus, which has been reported to stimulate feeding and GH secretion [36,37]. However, the interaction between ghrelin and other hypothalamic orexigenic peptides is complex and not totally clarified. Moreover, insulin can also stimulate hunger and food intake, acting in both a central and peripheral manner [38].

Being the action of cerebral hunger/satiety centers influenced by nutrients, it has been recently hypothesized that KDs could have a specific role in the regulation of appetite [39] (Table 1). In this regard, free fatty acids may provide a signal to the hypothalamus of nutrient abundance [40], and this may contribute to the appetite-reducing effects of KBs. In rodents, intra-cerebroventricular administration of a long-chain fatty acid markedly reduced food intake and hypothalamic expression of neuropeptide Y, a potent stimulator of appetite, whereas peripheral infusion of lipids, such as oleic acids, has been shown to reduce voluntary food intake in humans [40,41]. Noteworthy, Watanabe et al. reported that mice fed with a HFKD typically showed increased energy expenditure, though not changes in food intake [42].

Interestingly, a single meal rich in fatty acids and protein and poor in carbohydrates led to a differential postprandial secretion of appetite hormones, while visual analogue scales investigating appetite were similar to those recorded following a control meal [43]. A recent study by Tuccinardi et al. investigating the impact of walnut intake on food preference showed that walnuts consumption in the form of smoothies within a residential controlled setting led to increased serum BOHB and branched chain amino acids. Subsequent food intake, particularly protein and carbohydrate intake, was significantly decreased upon an ad libitum meal offered to the patients [44].

Although convincing, the bulk of evidence in relation to the inhibitory effects of ketosis on appetite is still anecdotal. Several studies have investigated the potential anorexigenic effect of the KDs, but the mechanisms of action of ketosis on appetite reduction are not completely understood yet. Clinical results are suggestive of both direct and indirect (via modifications of hunger-related hormones concentration) actions of KBs on appetite.

One of the proposed hypotheses according to which KBs may affect appetite is by acting on ghrelin. Moesgaard and colleagues shown that high-fat feeding lowers the expression of ghrelin in the stomach as well as the plasma level of ghrelin in C57BL/6J mice [45].

Two different studies showed reduced levels of ghrelin following the administration of ketone esters (KE) to healthy patients. The exact mechanism by which this occurs is not clear, though the concomitant reduction of GLP1 and PPY, or in any case a stability in the levels of these hormones, render the reduction of ghrelin more likely to be the main responsible for the changes in appetite [46,47]. In contrast, in a study by Sumithran et al., ghrelin levels remained constant and leptin levels decreased during ketosis, at which time patients reported a decreased sense of hunger. The absence of the ghrelin peak, associated with a decrease in appetite, reflects the strength of ghrelin as an orexigenic signal [48].

On the other hand, Boden et al., found higher ghrelin levels and decreased leptin levels in patients undergoing LCD which reported a preserved appetite but who still spontaneously reduced calorie intake. This suggests that the “satiating” effect of KDs goes beyond the conventional action of these hormones but is rather mediated by a concomitant reduction in insulin levels, observed in the study [47].

Putting ghrelin and leptin aside, Chearskul et al. hypothesized that the mechanism by which patients in a state of ketosis, reported less hunger is due to the fact that CCK and FFA levels were maintained at the same levels during ketosis as before the diet and then suffered a decline upon carbohydrates reintroduction, a period in which patients complained of a new increase in appetite [49].

Similar findings showed a spontaneous reduction in food intake during KDs, thought the exact mechanism was not investigated [50]. Johnstone et al. suggested that the difference between fat or carbohydrates intake could be the principal reason for less hunger in the study group, being the amount of protein intake equal with the control group [51].

Controversially, Nickols-Richardson imputed the higher protein amount as responsible for the reduction in hunger [52].

Additionally, it has been hypothesized a potential positive effect on mood exerted by KBs. In this regard, Burley et al. reported a reduction in hunger, irritability and urge to eat during the second week of VLCKD and the post diet week in eight obese women following VLCKD; while Johnston et al. reported a rise in satisfaction in 20 healthy man following a high protein-low fat diet [51,53].

Finally, Johnston et al., found no difference in hunger levels in obese patients following a LCKD vs. obese patients following a non-ketogenic diet, moreover the non-ketogenic group reported higher feeling of vigor-activity [51].

In summary, most of the studies in the literature on both humans and mice report that the ketogenic diet is associated with a reduction in appetite, probably mediated by an increase in ghrelin levels and a reduction in leptin levels. Similar results have been obtained following administration of KEs. Further studies are needed to assess the direct action of KBs on appetite.

### 3.3. Effect of the KD on Sleep Quality and Duration

As far as chrono-nutrition is concerned, one aspect that must be taken into consideration is the quality of sleep. Sleep–wake cycles influence circadian rhythmicity and feeding behavior. Different genes involved in metabolism show an altered transcription along with the modification of sleep patterns [54].

Clinical studies illustrated how circadian misalignment and sleep restriction were correlated with changes in glucose tolerance and insulin sensitivity, and therefore with a higher risk of developing diabetes and metabolic syndrome [55,56,57,58].

On the other hand, feeding behavior does influence sleep structure [59]. The consumption of certain nutrients and the adhering to certain dietary regimens can improve or disrupts sleep outcomes, probably due to a dietary influence on serotonin and melatonin activity. A study comparing the effect of carbohydrate-based meals with higher or lower glycemic index on sleep quality showed that the high-glycemic index meal resulted in a significant shortening of sleep onset latency and was most effective when consumed four hours before bedtime. This could be due to the fact that dietary carbohydrate intake increases the plasma concentration of tryptophan, a precursor of serotonin, and appears to induce sleep [60].

In this frame, we analyzed the role of ketosis in influencing sleep patterns (Table 2). Ketosis can follow a circadian rhythm, as proved by studies on rodents that showed how BOHB and BOHB dehydrogenase have the highest concentration during the night. These findings would explain their higher protective effects against seizure onset in the late night [61]. It is still not clear how the production of KBs affects sleep *per se* but different theories have been proposed. Adenosine, a purine with neuro-modulatory activity, has been reported to be a key player [62]. Adenosine acts at pre- and post-synaptic G protein coupled receptors and has inhibitory activity on neuronal excitability in different brain regions, thus promoting sleep by increasing its activity at the A1 and A2A receptors in the basal forebrain [63]. Ketogenic strategies increase ATP and other energy molecules in the brain, including extracellular adenosine. Furthermore, extracellular astrocyte-derived ATP appears to have a critical role in the sleep-promoting influence of adenosine [64].

Anatomically, the hypothalamus, with the ventro-lateral preoptic nucleus and the tubero-mammillary nucleus, withhold sub-regions that are specialized for the control of REM versus non-REM sleep. The neurons in these structures are primarily active during sleep, and produce the inhibitory neurotransmitters, galanin and GABA. Adenosine appears to increase the release of GABA at these levels and this increased release inhibits the histaminergic system and promotes sleep [65,66].

In light of these findings, the relationships among adenosine, GABAergic and galaninergic systems, KDs and sleep deserves to be further investigated.

Additionally, the role of leptin in KDs is being questioned. Due to its ability to interfere with the control of long-term energy homeostasis, it seems to represent an important molecular link between sleep, circadian rhythms, and energy metabolism. An impaired leptin signaling could have deleterious effects on sleep regulation: sleep amount, sleep architecture, and arousal stats appear to be affected, as reported by Laposky et al. [67].

In recent years, studies on rodents analyzed how fasting and the consequential increase of KBs affect sleep/wake patterns. The results suggest that PPARα and KBs may play important roles in the maintenance of wakefulness under conditions of lack of energy supply. Mice with PPARα deficiency that underwent food deprivation showed increased sleepiness and amount of NREM sleep [68]. In a previous study, mice treated with bezafibrate, a pan PPAR agonist, had increased slow wave activity in NREM sleep and a higher KBs ratio (AcAc/BHB) [69]. These findings were later confirmed in mice injected with AcAc and BHB that underwent sleep deprivation [70].

The clinical effects of the consumption of carbohydrates on sleep patterns are being studied. Back in 1975, Phillips et al. compared the effects of diets with different amounts of carbohydrates in eight healthy male and found a significant increase of REM sleep when the subjects were consuming the low-carbohydrate high-fat diet instead of the control isocaloric one [71]. Similarly, a significant increase in REM sleep was found by Kwan et al. in six women who underwent a 50-g carbohydrate, isoenergetic diet for a week [72].

Body composition, blood chemistry, and sleep abnormalities were evaluated in six adolescents suffering from obesity during and after an 8 week high-protein low-carbohydrate low-fat ketogenic diet. Beside a meaningful weight loss, the patients almost reached a normalization of sleep architecture, with a significant increase in REM sleep (from 13% to 21%, with an average for this age group of 21%) and a decrease in slow-wave activity (from 41% to 26%, with an average for this age group of 20%) [73].

Furthermore, Hallböök et al. reported preserved sleep structure in 18 children undergoing a HFKD for their therapy-resistant epilepsy. There was a significant decrease in total sleep and an increase in REM sleep, while slow wave sleep remained intact [74].

Afaghi et al. evaluated sleep response to an acute ketosis, after 48 h of very low carbohydrate meals [75]. Obviously, these finding are hard to compare to those of long-term treatment, such as patients with epilepsy. Further, it is not surprising that these findings differed from the previous ones: REM sleep was decreased, while slow wave sleep was improved. Some larger studies evaluated sleep disturbances only as possible collateral effects of KDs. For example, Guzel et al. analyzed how 389 children with drug resistant epilepsy reacted to an olive oil-based ketogenic diet and found that 20% of the patients lamented sleep disturbances during the first year of treatment [76].

Other studies concentrated on the effects of low-carbohydrate or KDs on altered sleep by addressing specific symptoms, such as sleepiness during the day. Nine patients with narcolepsy underwent a low-carbohydrate regimen for 8 weeks and showed a significant improvement of symptoms, with decreased sleepiness, sleep attacks, and sleep paralysis. A hypothesis is that this kind of diet induces a relative hypoglycaemia and therefore activates orexin containing neurons, that do improve daytime sleepiness [77].

Nineteen children with pyruvate dehydrogenase complex deficiency that underwent a ketogenic diet experienced improvement both clinically and in the development of motor and neurocognitive functioning. The diet was effective in reducing nocturnal awakenings and improving excessive daytime sleepiness [78].

Castro et al. evaluated 20 patients with obesity following a VLCKD and estimated sleep parameters, food and alcohol cravings, physical activity, sexual function, and quality of life. A significant improvement in sleepiness was observed but there was no difference in sleep quality and duration [79].

The effect of nutritional ketosis, reached with a three week-long isocaloric KD, on cognitive function were evaluated in 11 healthy patients in a randomized, crossover, controlled study. These patients also underwent a high-carbohydrates low-fat isocaloric diet. Sleep quality, morning vigilance, and mood were not different across dietary interventions [80].

Siegmann et al. conducted a non-randomized controlled study on 378 patients with type 2 diabetes and obesity. In a continuous care intervention, patients received individualized guidance to achieve nutritional ketosis. After one year, sleep quality was improved and the patients who were identified as “poor sleepers” at baseline (68.3%) were reduced to 56.5% [81]. A recent study evaluated the effects of HFKD on the quality of life of 29 women with breast cancer undergoing radiotherapy, considering sleep quality as one of the outcomes. In addition to sleep quality, the patients experienced significant improvements in emotional functioning, social functioning, future perspectives, and systemic therapy side effects (all *p*-values < 0.01) [82].

In brief, studies conducted so far show that a state of nutritional ketosis can significantly improve sleep quality, increase the REM sleep phase and reduce morning sleepiness. These results have been achieved with both HFKDs and VLCKDs and are likely to be attributable to the modulation of adenosine, GABAergic, and galaninergic systems by KBs.

### 3.4. Effect of the KD on Hormone Release

Among other physiological processes, endocrine body rhythms are tightly regulated by the circadian system. Consequently, the release of several hormones follows a circadian rhythmicity, and among these, cortisol, gonadal steroids, thyroid stimulating hormone (TSH), growth hormone (GH), and melatonin are the best characterized [83].

Some hormones released by the adrenal and pituitary glands show more activity at night. The most striking example is GH, the levels of which usually peak between 02:00 and 04:00 a.m. As a consequence, the disruption of the circadian rhythm (known as circadian dysrhythmia) can cause an improper secretion of GH [84].

Similarly, in humans, cortisol production usually increases during the night, reaching a peak of secretion just before waking in the morning, around 07:00 and 08:00 a.m. then declining throughout the day. Evidence suggests that cortisol can influence the expression of clock-controlled genes in the liver and adipose tissue [85].

Recent findings suggest that also TSH serum levels also have a strongly oscillatory profile, peaking between 02:00 and 04.00 a.m. and decreasing to its lowest levels between 04:00 and 08:00 p.m. In this regard, a recent review reported that the hypothalamic-pituitary–thyroid axis is under circadian oscillator control at multiple levels, from hypothalamic neurons to the thyroid gland [86].

The intrinsic rhythmicity of GnRH neurons suggests that these neurons may also express the components of the circadian oscillator, as demonstrated by Gillespie et al. Consequently, diurnal rhythms of pituitary gonadotropins, luteinizing hormone (LH) and follicle stimulating hormone (FSH), have been reported in women of reproductive age and are widely used to reflect the underlying characteristics of pulsatile GnRH secretion [87].

A nocturnal decline in serum LH levels in the early follicular phase of the menstrual cycle has been well established. However, it remains unclear whether these variations are a consequence of central circadian control from the suprachiasmatic nucleus or of external influences. In contrast, Klingman et al. reported no differences in LH pulse amplitudes between evening, night and morning, suggesting that endogenous circadian control does not contribute to previously reported diurnal rhythms [88].

Regarding serum total testosterone (TT), a circadian rhythm has been identified. TT rises at night, peaks between 07:00 and 10:00 a.m. and reaches its minimum at 07:00 p.m. [89].

In humans the normal daily prolactin (PRL) secretory profile displays a distinct circadian pattern characterized by a rise during nocturnal sleep and a rapid fall after awakening. This daily pattern is preserved during the menstrual cycle, although PRL levels are slightly higher in the luteal phase [90,91].

Finally, the study of human chronobiology has made enormous advances with the development of sensitive tests to measure melatonin, also known as the ‘mother hormone of chronobiology’, in plasma and saliva. Melatonin is the main hormone secreted by the pineal gland, starting soon after sundown, reaching a peak in the middle of the night and decreasing gradually during the second half of the night [92]. Receptors for melatonin are widely expressed in many peripheral and central nervous tissues. Therefore, the circadian rhythm of the pineal-derived circulating melatonin can have profound implications for the temporal organization of almost any organ [93].

The physiological effects of melatonin in regulating circadian rhythms are well known [94], and clinical trials also demonstrated its pharmacological positive application in circadian rhythm-related sleep disorders [95]. Noteworthy, preclinical evidence suggest that melatonin may affect feeding behavior [96]. In this regard, a recent study by Ríos-Lugo and colleagues in high-fat-fed rats showed that melatonin counteracts changes in hypothalamic gene expression of signals regulating feeding behavior [97]. Although no clinical study evaluated the relationship between KDs and melatonin, it is possible to speculate that KDs may reduce exogenous levels of this hormone, considering the low melatonin content in HFKD [98].

One of the most controversial topics surrounding the KDs is their safety as they relate to hormones. Over the last few years, there has been an increasing number of studies that investigate the short- and long-term effects of KDs on endocrine functions. Below we summarize all available clinical evidence regarding the impact of KD on the release of those hormones which follow a circadian pattern (Table 3). Among the various endocrine functions, most studies have evaluated the effects of the KDs in relation to the reproductive system.

Low carbohydrate diets have been shown to reduce insulin resistance [99]. Consequently, it has been proposed that KDs could have a role in the management of overweight and obese women suffering from polycystic ovarian syndrome (PCOS), a clinical condition which is often associated with hyperinsulinemia. In this regard, the putative endocrine effects of VLCKDs include improvements in body weight, free testosterone percentage, LH/FSH ratio and fasting insulin levels, as reported in a recent 24-week pilot study by Mavropoulos et al. [100]. These findings are in line with those reported by Paoli et al. who studied fourteen overweight women with a diagnosis of PCOS undergoing a ketogenic Mediterranean diet with phytoextracts for 12 week [26]. A different clinical trial concerned with reproductive health compared the efficacy of a structured exercise training programme against a hypocaloric hyperproteic non ketogenic diet in patients with PCOS. However, in this study, no significant change was detected in circadian hormones and metabolic parameters assessed [101].

To note, several studies investigated the role of KDs in the performance and body composition changes in men who practice regular physical activity. Intriguingly, Volek et al. reported that in athletes who have switched from a high-carbohydrate to a low-carbohydrate diet, long-term keto-adaptation resulted in extraordinarily high rates of fat oxidation [102]. As far as the effect of KD on TT serum level is concerned, a study by Wilson et al. reported a significant increase in TT in the group of training men undergoing an isocaloric KD compared to a traditional western diet (WD) [103]. Since T is synthesized from cholesterol, it can be assumed that a specific subgroup of KDs with a higher cholesterol content, the HFKDs, could result in increased TT levels. In this regard, Vidic et al. have recently demonstrated that an HFKD has the same impact as an high fat, low carbohydrate non-ketogenic diet on muscle strength, body-composition, and T profile in strength-trained middle-aged men [104]. As previously hypothesized, VLCKDs might have a minimal effect on T. In fact, another study evaluating the impact of a VLCKD on body composition and hormonal response did not show any significant changes in T or sex hormone-binding globulin [105]. In contrast, Mongioì et al. found an increase in TT and LH serum levels of 40 overweight/obese men who consumed VLCKD for at least eight weeks, suggesting that VLCKD is an effective, non-invasive and rapid tool to treat patients with obesity and concomitant metabolic hypogonadism [106]. In a study conducted on 36 22-day-old female Sprague–Dawley rats the administration of a HFKD decreased circulating concentrations of neuroactive steroids, including dihydrotestosterone, compared to a chow diet ad libitum [107].

Furthermore, the KDs mimic a state of starvation, determining a switch in metabolism from an anabolic to a catabolic state. Consequently, alterations in cortisol metabolism may be a short-term side effect of the KDs, as seen both in rodents and in humans. Interestingly, in a study conducted by Polito et al., morning salivary cortisol levels in 30 obese patients after 8 weeks of VLCKD were strongly decreased [108]. Similarly, Langfort and colleagues evaluated the effects of LCKD on metabolic and adrenal responses to graded exercise in men. In comparison with the normal diet, the KD resulted in lower plasma insulin concentrations and higher pre-and post-exercise levels of adrenaline, noradrenaline, and cortisol [109]. Moreover, these findings are in line with those of Stimson et al. who found that a 4-week ad libitum HFKD significantly affect cortisol metabolism independently of weight loss, enhancing cortisol regeneration by 11β-hydroxysteroid dehydrogenase type 1 [110]. Hainer et al. reported that a 4-week long VLCKD resulted in an improvement in cortisol diurnal rhythm with more pronounced suppressibility of morning cortisol by dexamethasone. Conversely, no changes in PRL secretion were recorded [111].

There is a small number of recent trials that have highlighted the effects of KDs on thyroid function. In general, diets with higher content of carbohydrates are associated with higher serum triiodothyronine (T3) concentrations. As it has been pointed out by Kaptein et al., similar to fasting, HFKD may significantly reduce T3 serum levels along with a concomitant increase in reverse-T3, and these changes are strictly correlated to the presence of KBs [112]. Little is known about the effects of KBs on the GH/IGF1 system. Bielohuby investigated how the administration of a HFKD for 4 weeks to 12-year-old Wistar rats could influence GH release. Interestingly, they found reduced hepatic GH receptor mRNA and protein expression, together with markedly reduced circulating insulin-like growth factor 1 (IGF1) and insulin-like growth factor 1 binding protein 3 (IGF1BP-3) [113]. Another study conducted on 22 children suffering from epilepsy demonstrated that HFKD could severely affect growth. In this regard, Spulber et al. found that serum IGF-I decreased immediately after the initiation of the KD and reached stable levels around 3 months after starting the diet. In particular, height velocity was most affected in those with pronounced ketosis, suggesting that the level of KBs should be related to outcomes both in seizure response and growth [114].

To summarize, numerous studies have evaluated the effect of nutritional chelation on hormone release. In particular, human studies have shown that following a reduction in carbohydrate consumption, the cortisol rhythm showed a phase delay and an increase in amplitude. Other authors have observed how the ketogenic diet in humans can lead to increased levels of total testosterone, TSH and LH, as well as a reduction in FT3, IGF-1, and the LH/FSH ratio. However, further studies investigating alterations in the daily rhythm of these hormones would be particularly useful in defining the role of ketone bodies in chronobiology.

## 4. Discussion

Metabolic homeostasis is strongly linked to circadian function and both central and peripheral clocks contribute to its maintenance. KDs are one of the candidate nutritional treatments to explore how specific diet-generated metabolites impact the peripheral clock.

Numerous strands of research have shown that both the composition of food and the timing of meals affect circadian rhythmic activity [115,116].

The crucial role of the timing of food intake is confirmed by the fact that eating at night is associated with increased body fat [117], insulin resistance and impaired weight loss [118]. Additionally, the KD appears to have a profound impact on circadian rhythm. Recently, an accurate review of the literature looked at the molecular mechanisms underlying the influence of KBs on chronobiology [119], pointing out that, in recent years, animal studies have been conducted to investigate the ability of KBs to modify circadian gene expression [120], and rearrange metabolic gene expression [19]. In particular, Gangitano et al. have described intriguing results regarding KD-induced modulation of clock gene expression in mouse models and sleep structure in humans. This review has elegantly illustrated how time of food intake and sleep all play roles in regulating circadian rhythm and that the KD can not only induce increased expression of certain clock genes, but can also result in *de novo* expression of oscillating genes in the liver [119]. However, the close connection between nutritional ketosis and circadian rhythms remains extremely complex and deserves further investigation, in relation to circadian processes including appetite, sleep–wake cycle, and hormone secretion.

KDs have proved to influence the peripheral clock in a tissue-specific manner in murine models [33]. Evidence from a clinical study evaluating an LCD suggests it could be the same in human subjects [121]. The ability of KD to modulate cyclic genes expression is partially attributable to beta-OHB non-canonical direct actions, which are independent of dietary total calorie intake [122].

**Figure 1:**
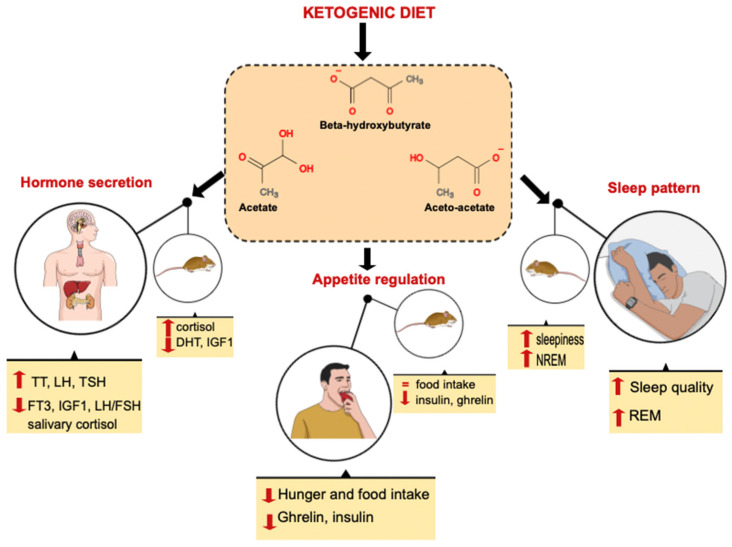
Evaluation of the effects of ketogenic diets on humans and mice in relation to three biological processes that follow circadian rhythms: appetite, sleep and endocrine function. Abbreviations: TT, total testosterone; LH, luteinizing hormone; FSH, follicle stimulating hormone; TSH, thyroid stimulating hormone; FT3, free triiodothyronine; IGF1, insulin-like growth factor 1; DHT, dihydrotestosterone; REM, rapid eye movement; NREM, non-rapid eye movement.

Our results suggest that KBs can have a specific role in the field of chrono-nutrition, influencing specific circadian processes, including appetite, sleep, and hormone release (Figure 1). The exact mechanisms through which KBs induce appetite suppression remains to be fully elucidated, since it reflects a complex interaction between peripheral and central signals produced by ketosis. Evidence suggests that ketones can act both increasing and reducing the sense of hunger. In addition, KBs are also implicated in the mechanism of food anticipation [123].

It can be postulated that the net balance of the contrasting stimuli results in a general reduction of perceived hunger and food intake, however more studies are needed to explore the mechanism of potential beneficial effects of KDs on food control. Understanding how to take full advantage of the appetite suppressant action of KBs, could be an extremely valid help to achieve, and maintain, weight loss in patients with obesity.

The studies conducted up to this point do not allow us to determine if KDs improve sleep parameters and overall sleep quality. It is difficult to compare the different studies because they have been conducted in very different settings. Most of these that analyze long term KDs are conducted on children with epilepsy, and these conditions may lead to a series of biases when evaluating sleep parameters. First, the different sleeping habits and sleep characteristics of children may not be comparable to those of adults. In dealing with epilepsy, most studies choose a high-fat low-carbohydrate diet, but each kind of diet, with various timings, does offer different chances to reach ketosis, so the effects of KBs themselves are not always measurable. When analyzing patients with different basal conditions, the effects of the diet on the pathology itself may represent a bias. For example, the efficacy of KDs on improving seizure could affect the quality of sleep in patients with epilepsy, who would experience less nocturnal awakenings and less daytime sleepiness. In patients with obesity, the weight loss induced by the diet improves sleep apnea, a very common comorbidity in these patients, hence improving sleep quality and sleepiness as well [58].

Sleep parameters were not analyzed in the same way in all studies: some conducted EEG at different timings, allowing us to identify REM and NREM patterns, while others only evaluated sleep with questionnaires on perceived sleep quality. Despite all these underlying conditions, some results are consistent and promising and the KDs appear to be a safe option in different settings and do not seem correlated with a negative impact on sleep outcomes. However, there is not enough evidence on its capability to improve sleep, especially in comparison with other dietary regimens. Further controlled studies with larger samples, and aimed to analyze sleep in its different components, are needed in order to better identify the role played by ketosis and the KD.

As far as hormone regulation in concerned, there are two different currents of thought in relation to the KDs. The first states that ketosis is a stressor, mimicking starvation, while the second hypothesis assumes that ketosis is natural and safe by reproducing the dietary lifestyle before modern development. Several findings have shown that KBs can significantly influence hormone release both in mice and in humans, but specific studies with serial sampling are needed to assess how the KDs can modulate the circadian rhythm of hormones.

## 5. Conclusions

This review is a summary of available data on the effectiveness of KDs in the regulation of circadian processes. KBs are candidate elements to explore how metabolites generated by specific diets affect the peripheral biological clock. Specifically, KBs have been shown to affect the peripheral clock in a tissue-specific manner in mouse models. [33] Recent strands of research suggest that these events could also occur in humans. The ability of KBs to modulate cyclic gene expression is partially attributable to their non-canonical direct actions, independent of total caloric dietary intake.

## Figures and Tables

**Table 1:** Selected studies regarding the effect of a ketogenic diet or ketone bodies on appetite regulation.

First Author	Year	Study Design	Species	Duration	Number of Subjects n (f)	Type of Diet	Control	Results
Burley	1992	CT	Humans	2 wk	8 (0)	VLCKD	none	↓ hunger, ↓ irritability, ↓ urge to eat during the second week of VLCKD and the post-diet week
Boden	2005	CT	Humans	2 wk	10 (7)	HFKD	none	spontaneously ↓ energy intake, =level of hunger and satisfaction, ↓leptin, ↑ghrelin, ↓insulin
Chearskul	2008	CT	Humans	8 wk	12 (0)	VLCKD	none	=levels of CCK and FFA during ketosis,↓ postprandial CCK and FFA concentrations after weight loss. ↓ hunger during ketosis, =at week 0 and 1 week after diet
Vestergaard	2021	RCT	Humans	Single adm	10 (0)	KE	placebo, glucose	↓ hunger and prospective food consumption, ghrelin and GLP-1
Sumithran	2013	CT	Humans	8 wk	39 (n.a.)	VLCKD	none	during ketosis = ghrelin, ↓ leptin and subjective ratings of appetite
Johnston	2006	RCT	Humans	6 wk	19 (15)	VLCKD	non ketogenic low carbohydrate (157 g)	=hunger
Johnstone	2008	RCT	Humans	4 wk	17 (0)	HFKD	medium carbohydrate intake non ketogenic diet (35% carbohydrate)	↓ hunger and food intake
Nickols-Richardson	2005	RCT	Humans	6 wk	28 (28)	HFKD	high-carbohydrate/low-fat	↓ hunger
Johnston	2004	RCT	Human	6 wk	16 (n.a.)	HPLFKD	high-carbohydrate/low-fat (280 g)	↑ satisfaction
Vancy	2004	RCT	Humans	24 wk	79 (n.a.)	HFKD	low fat diet	↓ energy intake
Stubbs	2017	RCT	Humans	Single adm	15 (5)	KE	isocaloric dextrose	↓ of hunger and plasma insulin, ghrelin, GLP-1, and PYY levels

Abbreviations: CT, clinical trial; RCT, randomized control trial; VLCKD, very low calorie ketogenic diet; HFKD, high-fat ketogenic diet; KE, ketone ester; CCK, cholecystokinin; FFA, free fatty acid; GLP-1, glucagon-like peptide-1; PYY, peptide YY; HPLF, high protein low fat diet; HFKD, high fat ketogenic diet; adm, administration; g, grams.; wk, weeks; n.a., not available.

**Table 2:** Selected studies regarding the effect of a ketogenic diet on sleep pattern.

First Author	Year	Study Design	Species	Duration	Number of Subjects n (f)	Type of Diet	Control	Results
Phillips	1975	RCT	human	4 d	8 (0)	low-carbohydrate high-fat diet	HCLFD	↑ REM sleep
Kwan	1986	CT	human	1 wk	6 (6)	low-carbohydrate isoenergetic diet	none	↑ REM sleep
Willi	1998	CT	human	8 wk	6 (3) adolescents with obesity	HPLFKD	none	↑ REM sleep, ↓ SWS
Husain	2004	CT	human	8 wk	9 (1) with narcolepsy	LCD	none	improve sleepiness, sleep attacks and sleep paralysis
Hallböök	2007	CT	human	3–12 m	18 (9) children with therapy-resistant epilepsy	HFKD	none	↓ total sleep, =SWS, ↑ REM sleep, ↓ sleep stage 2, =stage 1
Afaghi	2008	CT	human	48 h	14 (0)	LCD	none	↑ SWS, ↓ REM
Sofou	2017	CT	human	6 m–6 y	19 (16) children with PDC deficiency	KD	none	improve nocturnal awakenings and daytime somnolence
Guzel	2018	single-center, prospective study	human	3–24 m	389 (187) children with drug-resistant epilepsy	olive oil-based KD	none	sleep disturbances in 20%
Castro	2018	LCT	human	60–90 d	20 (12) with obesity	VLCKD	none	improvement in sleepiness, =sleep quality, =duration
Iacovides	2019	RCT crossover,	human	3 wk	11 (10)	HFKD	HCLFD	=sleep quality, =morning vigilance
Siegmann	2019	NRC longitudinal study	human	n.a.	378 (259) with T2D and obesity	VLCKD	none	↑ sleep quality
Klement	2021	CT	human	n.a.	29 (29) women with early-stage breast cancer undergoing radiotherapy	HFKD	none	↑ sleep quality

Abbreviations: LCT, longitudinal clinical trial; RCT, randomized control trial; CT, clinical trial; n, numbers; f, females; d, days; wk, weeks; m, months; y, years; National Research Council; T2D, type two diabetes; REM, rapid eye movement; SWS, slow wave sleep; n.a., not available; KD, ketogenic diet; HFKD, high-fat ketogenic diet; VLCKD, very low-calorie ketogenic diet; LCD, low carb diet; NRC, HPLFKD, high protein low fat ketogenic diet; HCLFD, high carb low fat diet.

**Table 3:** Selected studies regarding the effect of a ketogenic diet on hormone release.

First Author	Year	Study Design	Species	Duration	Number of Subjects n(f)	Type of Diet	Control	Results
Mavropoulos	2005	LCT	Human	24 wk	11 (11)	VLCKD	none	↓ percent FT (−22%), ↓ LH/FSH ratio (−36%)
Wilson	2020	RCT	Human	10 wk	25 (0)	Isocaloric KD	WD	↑ TT
Paoli	2021	RCT	Human	8 wk	19(0)	VLCKD	WD	↓ TT and IGF1
Mongioì	2020	LCT	Human	at least 8 (13.5 ± 0.83 wk)	40 (0)	VLCKD	none	↑ TT and LH
Rhodes	2005	RCT	Dawley rats	6 wk	36 (36)	KD	ad libitum chow	↓DHT
Vidić	2021	RCT	Human	8 wk	20 (0)	HFKD	iso energetic NKLCHFD	↑ TT and FT
Kose	2017	CT	Human	14.7 ± 3.6 wk	120 (57)	HFKD	none	↑ TSH, ↓ FT3, ↓ FT4
Iacovides	2018	RCT	Human	ongoing	40 (20)	VLCKD	HCLF	ongoing
Volek	2002	CT	Human	6 wk	12 (0)	VLCKD	ND	↑ FT4; ≈TT, FT, SHBG, cortisol or FT3
Kaptein	1985	LCT	Human	40 d	10 (0)	HPKD	none	≈TSH, ↓ FT3, ↑ FT4, ↑rT3
Van Dam	2002	LCT	Human	7 d	15 (15)	VLCKD	none	↑ LH
Paoli	2020	PCT	Human	12 wk	14 (20)	LCKD	none	↓ LH/FSH ratio, ↓FT, ↑estradiol, ↑progesterone, SHBG
Langfort	1996	CT	Human	3 d	8 (0)	HFKD	mixed diet	↑ cortisol
Stimson	2007	RCT	Human	4 wk	17 (0)	HFKD	MFMCD	↑ cortisol; ↓ 5alpha- and 5beta-reduced 24H-cortisol metabolites
Bielohuby	2011	3 arms CT	Wistar rats	4 wk	16 (0)	HFKD	normal CH and LCNKD	↓ IGF1, ↓ GHR, ↓ IGFBP-3
Spulber	2009	LCT	Human	3–6 m	22 (9)	LCKD	none	↓ IGF1

Abbreviations: LCT, longitudinal clinical trial; RCT, randomized control trial; CT, clinical trial; n, numbers; f, females; d, days; wk, weeks; m, months; KD, ketogenic diet; HFKD, high-fat ketogenic diet; VLCKD, very low calorie ketogenic diet; LCD, low carb diet; WD, western diet; HPKD, high protein ketogenic diet; CH, carbohydrates; LCNKD, low carb non ketogenic diet; FT, free testosterone; TT, total testosterone; LH, luteinizing hormone; FSH, follicle stimulating hormone; TSH, thyroid stimulating hormone; FT3, free triiodothyronine; FT4, free thyroxine; SHBG, sex hormone binding globulin; r-T3, reverse-T3; GH, growth hormone; IGF1, insulin-like growth factor 1; IGF1BP-3, insulin-like growth factor 1 binding protein 3.

## Data Availability

Data will be made available upon reasonable request to the corresponding author.
